# QSAR Development for Plasma Protein Binding: Influence of the Ionization State

**DOI:** 10.1007/s11095-018-2561-8

**Published:** 2018-12-27

**Authors:** Cosimo Toma, Domenico Gadaleta, Alessandra Roncaglioni, Andrey Toropov, Alla Toropova, Marco Marzo, Emilio Benfenati

**Affiliations:** Laboratory of Environmental Chemistry and Toxicology, Department of Environmental Health Sciences, Istituto di Ricerche Farmacologiche Mario Negri IRCCS, Via la Masa 19, 20156 Milano, Italy

**Keywords:** ADME, fu, logk, protein binding, QSAR

## Abstract

**Purpose:**

This study explored several strategies to improve the performance of literature QSAR models for plasma protein binding (PPB), such as a suitable endpoint transformation, a correct representation of chemicals, more consistency in the dataset, and a reliable definition of the applicability domain.

**Methods:**

We retrieved human fraction unbound (Fu) data for 670 compounds from the literature and carefully checked them for consistency. Descriptors were calculated taking account of the ionization state of molecules at physiological pH (7.4), in order to better estimate the affinity of molecules to blood proteins. We used different algorithms and chemical descriptors to explore the most suitable strategy for modeling the endpoint. SMILES (simplified molecular input line entry system)-based string descriptors were also tested with the CORAL software (CORelation And Logic). We did an outlier analysis to establish the models to use (or not to use) in case of well recognized families.

**Results:**

Internal validation of the selected models returned Q^2^ values close to 0.60. External validation also gave r^2^ values always greater than 0.60. The CORAL descriptor based model for √*fu* was the best, with r^2^ 0.74 in external validation.

**Conclusions:**

Performance in prediction confirmed the robustness of all the derived models and their suitability for real-life purposes, i.e. screening chemicals for their ADMET profiling. Optimization of descriptors can be useful in order to obtain the correct results with a ionized molecule.

**Electronic supplementary material:**

The online version of this article (10.1007/s11095-018-2561-8) contains supplementary material, which is available to authorized users.

## Introduction

Drugs can form reversible bonds with plasma proteins, heavily influencing the pharmacological response. Only the free concentration of the drug in tissues guarantees the biological effect. The pharmacokinetic behavior is very important, and in the last few years almost 10% of failures in drug development have been due to this reason (1).

Drug absorption is very sensitive to plasma protein binding (PPB). Small changes in the fraction bound to proteins can have a significant impact on the bioavailable fraction of the drug and this influence is even more obvious when large fractions are bound. A difference between 98% and 99% of bound drug results in double the amount of drug available in plasma even though such a small difference may not appear significant. This implies a narrower therapeutic index and a longer half-life of mostly bound drugs compared to others (2).

Plasma is the principal component of human blood (55%) and it is made up of water (92%), proteins (7%) and other solutes (1%). Albumin is the protein with the highest concentration in plasma, followed by globulins, clotting factors and regulatory protein. Most drugs bind with specific proteins, whether they act as acidic or basic compounds, and have different binding sites on the same plasma protein. Generally speaking, acidic compounds bind with albumin and basic compounds with lipoproteins and α1-acid glycoprotein (2).

For this study we used a collection of *in vivo* PPB values, but in recent years several *in vitro* techniques have been developed (2). Some have also been used for estimating the binding to a specific protein, e.g. albumin (3). However*, in vitro* and *in vivo* methods are often expensive and demanding in terms of time and resources (e.g. reagents and detection techniques).

A quantitative structure-activity relationship (QSAR) is defined as “an equation or other function that describes the relationship between a biological property of compounds, usually a measure of relative potency” namely an endpoint, “and one or more properties of the compounds”, (4). Ideally the endpoint refers to a single mechanism of action, but this is not the case of PPB. Drugs can bind different plasma proteins, and the same protein (especially albumin) can have different binding sites. Therefore it is not easy to establish a universal model (5). However, some properties such as lipophilicity are important in PPB, with no specific relation to a single plasma protein. This makes possible to identify common quantitative parameters relevant for QSAR (2).

QSAR models are also influenced by quality of the dataset. PPB data show intrinsic variability due to the use of different methods, experimental conditions or endpoint transformations. Several *in silico* models have been developed, with different data sets and different measurement units. In this regard, *in silico* methods can be cheap, rapid and powerful for screening large quantities of chemicals, even without the need for the substance to be synthetized, because its structure is sufficient. Looking at QSAR models in the literature, there is a wide range of data sources, structure representations, descriptors, learning algorithms, and validation criteria (6). A starting point in dataset building for many PPB models is Goodman and Gilman’s book *Pharmacological Basis of Therapeutics* (7), a solid collection of data retrieved from the literature.

Various efforts have been made to integrate new data, often starting from *in vitro* or interspecies analysis, or from data calculated from other pharmacokinetic parameters via differential equations. However, the use of calculated data may lead to a decrease in the quality of the final dataset. Various modeling approaches have been used too, and different data representations (e.g. fraction unbound (*fu*), fraction bound (*fb*), percent bound (%PPB), pseudo-equilibrium constants such as logK, lnKA, etc.) have been used to improve performance. The best results were obtained with boosted regression tree, random forest, partial least squares, support vector machine (SVM), k-nearest neighbor (k-NN), heuristic algorithm (HA).

Other studies focused on albumin serum affinity (HSA) with methods as SVM or HA (8) or tried to integrate QSAR and docking scores (9), including geometry optimization before modeling to improve performance (10).

The aim of this study was to evaluate the influence of some key parameters such as different molecule representations, endpoint transformations, modeling algorithms and applicability domain (AD) definitions. In addition, the models were evaluated for suitability on specific families of chemicals.

## Materials and Methods

### Data Curation

Data from Obach *et al.* (11) were used for modeling. This is a collection of human *fu in vivo* data (670 compounds) retrieved from the literature, related mostly to drugs. The compounds without experimental values or those with values expressed as a range were eliminated. SMILES were automatically retrieved using chemical name and chemical abstract service (CAS) as identifiers. JChem and Chemcell (12) were used for retrieving SMILES. Compounds with missing SMILES or incongruences between the two sources were discarded.

Chemicals were neutralized and counter-ions eliminated too. Substances with ambiguous information, metal complexes and inorganic compounds were eliminated. After this cleaning process, the final dataset comprised 512 compounds.

**Fig. 1:**
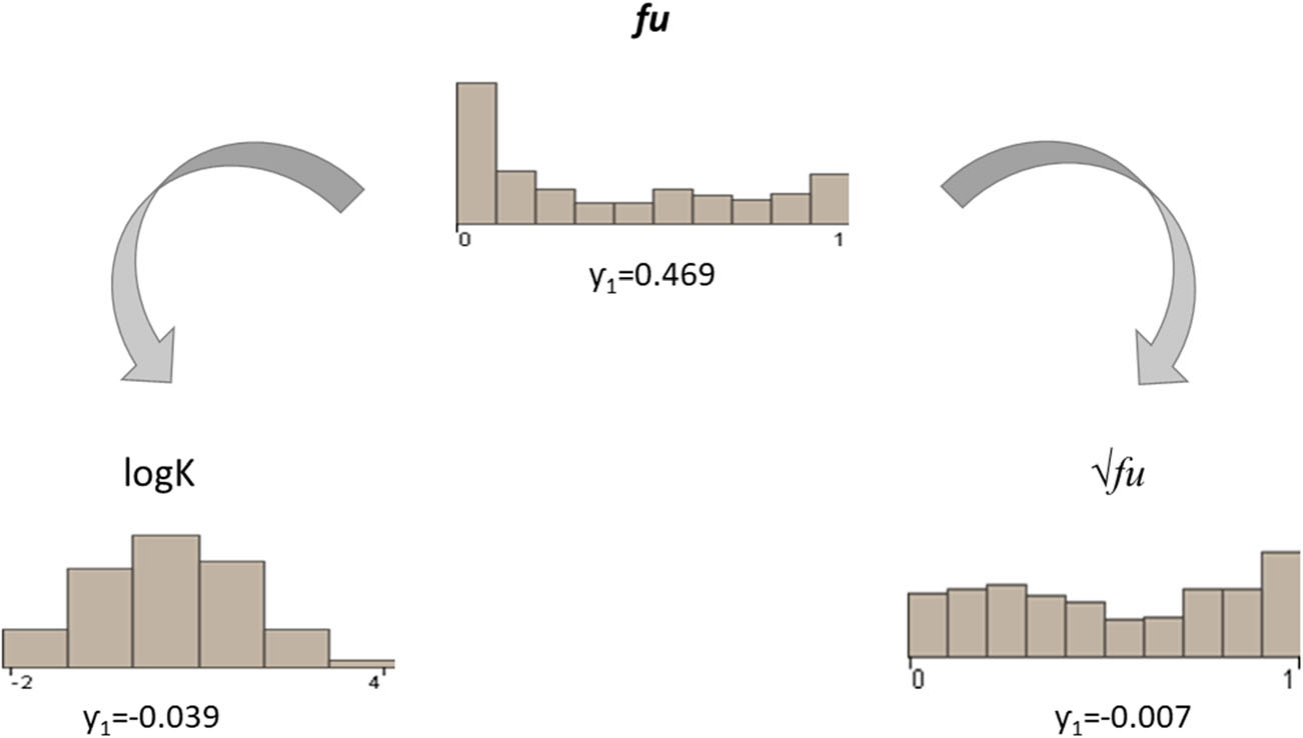
Representation of the distribution of PPB data, from Obach (13), before and after transformation. The γ_1_ of each distribution is indicated.

The first issue to face was the skewness (*γ*_1_) of the data set: the distribution of experimental values was shifted toward low values. A significant part of the dataset consisted of compounds with a highly bound with proteins, with values between 0 and 0.1 (see Fig. 1). The first bar of the histogram in the upper part of Fig. 1 is much higher than the others, and usually compounds in this activity range are those with a narrower therapeutic index. In order to derive a model able to discriminate small differences in activity and to obtain a distribution more suitable for modeling, we applied two different endpoint transformations.

The first transformation is a pseudo equilibrium constant (3,5,6,14) expressed as in Eq. 1:

1 $$ \mathrm{logK}=\log \left(\frac{1- fu}{fu}\right) $$

When *fu* is equal to 100%, logK is arbitrarily set at 2.

The second transformation is the square-root of *fu* ($\sqrt{fu}$).

Figure 1 shows the distributions of values before and after the transformations with the relative γ_1_ value. As expected, logK and *√fu* had less skewed distribution, making them more suitable for modeling than the original *fu* data.

### Model Derivation

We used two approaches to obtain QSAR models for PPB. The first applies machine learning algorithms on molecular descriptors based on chemical features of the compounds. The second approach used CORAL (IRFMN, 2017) software which implements a descriptor extraction algorithm from a SMILES string.

### Calculation of Molecular Descriptors

The main (de)protonated form of the molecule on the dataset at physiological blood pH (7.4) was determined with JChem (15). SMILES were modified accordingly. Dragon 7.0 (16) was used to calculate 2D molecular descriptors. Dragon was not able to calculate several descriptors for 23 compounds. Due to the importance of some of these descriptors (for instance AlogP) we decided to exclude these compounds instead of reducing the number of predictors of the model.

Many of the Dragon descriptors are likely to be redundant or not informative, adding uncertainty to the model and lowering its effectiveness (17), besides the longer computational time needed. Although some models are naturally resistant to non-informative predictors, it is obvious that reducing the input space is an important step in model derivation. For this reason, descriptors with constant values (standard deviation 0) and descriptors that correlate over 95% (Pearson correlation coefficient) with another were rejected. Variable selection was then applied using a random forest based approach as implemented in (18) package for R. It is based on three steps. The first iterates a series of random forests, then the algorithm calculates the variable importance (based on permutation score) and eliminates those variables that fall below a user-defined threshold. The second step finds important descriptors closely related to the response variable (interpretation step) and the third step (prediction) identifies the smallest model leading to a good prediction of the response variables.

As the ionization state is important in determining PPB, local models for specific protonation states (acids, bases, neutral chemicals and zwitterions) were also derived. We used ACD/labs 12.0 to calculate the concentration of (de)protonated molecules at pH 7.4. If a molecule is more than 10% in the acid or basic state, it is flagged as acid or base; if a molecule is more than 10% for both the acid and the base ionization state, it is considered a zwitterion. Neutral substances have more than 90% of the concentration in a neutral state. The number of chemicals in each dataset is shown in Table I.

**Table I:** Compounds in Each Datasets for Specific Ionization States

Ionization state	No. compounds
Acid	122
Base	137
Neutral	198
Zwitterions	55
Total (used for modelling)	489

When addressing the four subsets with specified ionization states, the neutral form of the molecule was used to calculated Dragon descriptors (since the ionization state was homogeneous in each subset).

For this reason, we were able to save all the compounds for the local models.

When modeling the sub-datasets, the square roots of the fraction unbound gave a better performance, so only these results are shown.

### Data Splitting

For the model’s derivation, the dataset was divided into a Training Set (TS) and an External Validation Set (EVS) with a ratio of 80:20. The number of compounds in each set is shown in Table II. In order to ensure a uniform distribution of the endpoint values in the two subsets, we applied an activity sampling method. The dataset was binned into five equal sized portions based on fixed ranges of experimental values. Each bin was then divided based on a 80:20 ratio and then distributed in TS and EVS .

**Table II:** Numerosity of the Splits for Each Dataset and Number of Descriptors Selected

Transformation	No. of selected Dragon Descriptors with VSURF	No. Of compounds in TS	No. Of compounds in EVS
Total LogK	24	391	98
Total √fu	16	391	98
Acids √fu	8	97	25
Base √fu	18	158	40
Neutral √fu	10	109	28
Zwitterions √fu	6	47	8

### Model Training

After VSURF variable selection, a Random Forest (19) algorithm, as implemented in KNIME (20) was applied for model derivation. Data sampling for each tree was done with replacement, and the default number of randomly chosen descriptors at each split was set as the square root of the initial number of descriptors; the descriptors are different for each tree.

### Applicability Domain

The AD of a QSAR model is defined as “the physico-chemical, structural, or biological space, knowledge or information on which the TS of the model has been developed, and for which it is applicable to make predictions for new compounds […]. Ideally, the QSAR should only be used to make predictions within that domain by interpolation not extrapolation” (21).

Since there is not a universally accepted method to define AD (21–23) a series of approaches were applied. Results were evaluated in terms of gain in performance resulting from the removal of prediction out of AD, and coverage (percentage of chemicals retained after the application of a given AD method) (Table III).

**Table III:** Methods Chosen for Defining the AD, Brief Description and Reference

Method	Description
Two-class real-random classification	After permutation of descriptors on a mirror TS, the two matrices are merged and a classification model is built to distinguish real values from random ones. (17,24)
Leverage	Based on calculation of the leverage (hi). New compounds that are above the hi threshold are considered outside the AD. (25,26)
PCA (threshold: mean±3*SD)	After calculation of the two first PC of TS descriptors a threshold is set for each PC equal to mean ± 3*standard deviation. If values for PCs of new compounds fall outside the established range, the prediction is considered unreliable. (23)
PCA (threshold: 0.5-0.95 percentile)	Same as the method above, but the threshold is established on the 0.5th and 0.95th percentile of distribution of TS compounds. (23)
Nearest neighbor distance	It is based on calculation of the average Euclidean distances between all pairs of TS compounds. If the distance of a VS compound from its nearest neighbor in TS is greater than a given threshold, it is out of AD. (27,28)
Atom centered fragment (ACF)	All ACFs are calculated (a central non-hydrogen atom with all atoms bonded to it) of the TS. A test compound is considered within the AD if each ACF obtained by its decomposition is part of the ACFs identified in the TS. (29–31)
Fingerprint	The average similarity (Tanimoto based on PubChem fingerprints) of test compounds with the TS is determined. If average similarity is lower than 0.1 the compound is outside the AD. (23)

### SMILES-Based Descriptors Model Derivation (CORAL)

The optimal descriptors calculated with CORAL (http://www.insilico.eu/coral/) software are attributes extracted from parsing the molecule’s SMILES notations. Obviously the most important treatment in this case is the correct normalization of the SMILES notation because the algorithm works by recognizing recurrent patterns (particular characters or combinations) in the SMILES (32–34). To have a good standardization of patterns the SMILES notation has been canonicalized with ACD/labs (35). The possible SMILES attributes are listed in Table IV.

**Table IV:** Smiles Attributes and their Description

SMILES attibutes	Description
*Sk*	*Single SMILES element*
*SSk*	*Combination of two SMILES elements*
*SSSk*	*Combination of three SMILES elements*
*HARD*	*Represents the presence, or absence of eight chemical elements (nitrogen, oxygen, sulfur, phosphorus, fluorine, chlorine, bromine, and iodine) and different kinds of chemical bonds (double bond, triple bond, and stereo chemical bond)* (36)*.*

The TS used for Dragon approach modeling has been further divided into three sets: a TS of 108 compounds, an Invisible Training Set (ITS) of 140 compounds, a Calibration Set (CS) of 143 compounds. Conversely, the validation set is identical to the EVS used with the Dragon descriptor-based models.

The endpoint is calculated as in Eq. 2:

2 $$ \mathrm{Endpoint}={\mathrm{C}}_0+{\mathrm{C}}_1\ \mathrm{DCW}\left(\mathrm{T},\mathrm{N}\right) $$

C_0_ and C_1_ are the intercept and slope for the Eq. 2, and *DCW(T, N)* is the combination of SMILES-based attributes, each associated with a correlation weight (*CW*), as described in Eq. 3. The correlation weights are optimized with the Monte Carlo method to  a given number of iterations (N), providing *CWs* which, used in Eq. 4, provide a maximum correlation coefficient between the descriptor and selected endpoint.

3 $$ DCW\left({T}^{\ast },{N}^{\ast}\right)= CW(HARD)+\sum CW\left({S}_k\right)+\sum CW\left({SS}_k\right)+\sum CW\left({SS S}_k\right) $$

The *CW(HARD)* is the correlation weight of the *HARD*.

The *S*_*k*_ is the SMILES atom (i.e. single symbol or two symbols which cannot be examined separately, e.g. ‘Cl’, ‘Br’, etc.); the *SS*_*k*_ is a combination of two SMILES atoms; the *SSS*_*k*_ is a combination of three SMILES atoms. The *CW(S*_*k*_*)*, *CW(SS*_*k*_*)*, and CW(SSS_*k*_*)* are correlation weights of the above SMILES attributes. The numerical data on the correlation weights are calculated by the Monte Carlo method. The optimization gives maximal value for target function. The target function (*TF*) is calculated as Eq. 4:

4 $$ TF=R+{R}^{\prime }-\left|R-{R}^{\prime}\right|+ IIC\ast 1.1 $$

R and R’ are the correlation coefficients between experimental and predicted values of the endpoint for TS and ITS, respectively. The *IIC* is the Index of Ideality of Correlation described in the literature (37,38). Attributes with positive *CW* are considered promoters of an increase of the endpoint value, and those with negative *CW* are promoters of a decrease. CORAL has an in-house AD evaluation. Only compounds whose SMILES attributes have been selected for model derivation are considered in AD. Predictions of chemicals outside the model AD are considered unreliable and with greater uncertainty and are excluded from the evaluation of the performance (39).

### Statistical Analysis

Performance is evaluated on the basis of the determination coefficient (r^2^) calculated as shown in Eq. 5.

5 $$ {\mathrm{r}}^2=1-\frac{\sum {\left({\mathrm{y}}_{\mathrm{i}}-{\hat{\mathrm{y}}}_{\mathrm{i}}\right)}^2}{\sum {\left({\mathrm{y}}_{\mathrm{i}}-{\overline{\mathrm{y}}}_{\mathrm{i}}\right)}^2} $$

where y_i_ is the experimental value of the i-th chemical in the dataset; ŷ_i_ is the predicted value of the i-th query compound in the dataset for the determination of r^2^; ${\overline{\mathrm{y}}}_{\mathrm{i}}$ is the mean of the experimental values of the compounds in the dataset.

Root Mean Square Error (RMSE) is the square root of the average of the squared differences between prediction and actual observation, as represented in Eq. 6:

6 $$ \mathrm{RMSE}=\sqrt{\sum \frac{{\left({\hat{\mathrm{y}}}_{\mathrm{i}}-{\mathrm{y}}_{\mathrm{i}}\right)}^2}{\mathrm{N}}} $$

where y_i_ is the experimental value of the i-th chemical in the dataset; ŷ_i_ is the predicted value of the i-th chemical and N is the number of chemicals.

The cross-validated determination coefficient (Q^2^) has been used for the calculation of statistics in cross-validation.

7 $$ {\mathrm{Q}}^2=1-\frac{\sum {\left({\mathrm{y}}_{\mathrm{i}}-{\overset{\prime }{\mathrm{y}}}_{\mathrm{i}}\right)}^2}{\sum {\left({\mathrm{y}}_{\mathrm{i}}-{\overline{\mathrm{y}}}_{\mathrm{i}}\right)}^2} $$

${\overset{\prime }{\mathrm{y}}}_{\mathrm{i}}$ is the predicted value in cross-validation (40).

For the Dragon models a 5 fold internal cross-validation (5-fold cv) is used while in the case of CORAL model the equation is calculated as the aggregation of TS, ITS and CS.

### Outlier Analysis

A statistical analysis was done in order to check for the possible presence of chemical categories with a large error in prediction. Compounds with absolute error in prediction larger than the mean absolute error (MAE) observed for the whole TS were considered badly predicted (outliers); the remaining compounds were considered correctly predicted.

Chemical categories were defined based on the occurrence in their structures of some “Functional group count” descriptors calculated by Dragon 7.0 (16). Then the distribution  of outliers in each category is compared with the distribution of outliers of the entire dataset by a significance test (Fisher’s exact test). This statistic tests the null hypothesis if there is no association between the row variable and the column variable. In this particular case the null hypothesis is the absence of significant difference from the distribution of outliers in a category and in the total distribution. The null hypothesis is rejected when the p-value is less than 0.05.

To evaluate the strength of the probability Likelihood Ratio has been adapted from Ferrari *et al.* (41) to estimate the statistical relevance of analyses. (Eq. 8)

8 $$ \mathrm{LR}={\left(\mathrm{TP}/\mathrm{FP}\right)}^{\ast}\left(\mathrm{negatives}/\mathrm{positives}\right) $$

The TP (true positives) are compounds with a certain functional group that are badly predicted, while the FP (false positives) are compounds with the same functional group but correctly predicted. Negatives are the total number of correctly predicted compounds, while positives are the total number of badly predicted compounds.

The same procedure has been used also to evaluate if some of the models performed better for certain chemical categories.

## Results

Table V shows that the statistical performance of the various models is comparable. Internal validation returned Q^2^ values close to 0.60 for Dragon and CORAL models. External validation also gave r^2^ values around 0.71, with the CORAL model performing better than others, with a r^2^ value of 0.74 on the *VS.* LogK model gave the best performance when PCA based AD (threshold: mean±3*SD) was used, while √*fu* model had the most noticeable improvements when Two Class Real-Random Classification based AD was applied. Few chemicals (between 3% and 13%) were excluded after AD application when we focus on the Dragon models, while CORAL model has a lower coverage.

**Table V:** Performance of PPB Predicting Models

	r^2^/Q^2^	RMSE	Coverage	AD
Dragon(log K)				
TS (5-FOLD CV)	0.61	0.72	-	
EVS	0.65	0.68		
EVS (in AD)	0.68	0.65	0.98	PCA – mean±3*SD
Dragon(*√fu*)				
TS (5-FOLD CV)	0.62	0.19	-	
EVS	0.70	0.17		
EVS (in AD)	0.72	0.16	0.87	Two-class Real-Random Classification
CORAL (*√fu*)				
TS+ITS+CS	0.61	0.19	-	
EVS	0.69	0.17		
EVS (in AD)	0.74	0.12	0.77	CORAL AD

RMSE values of logK model and √*fu* model cannot be compared, as the two endpoints differ in their spread of experimental values. Performance was acceptable in both internal and external validation, while excluding compounds out of AD slightly improved performances without losing too much in coverage. The internal validation for the Dragon models is performed with a 5-fold cross-validation.

It is not simple to generate valid models for compounds discriminated on the basis of their (de)protonation state. The use of ionized state did not improve performance, so we used the classical SMILES notation.

Among the models for specific protonation states, only the model for acid compounds gave acceptable performance in both internal and external validation, while other models gave disappointing results in external validation raising to acceptable results only if the compounds were included in the AD but resulting in a very large decrease in coverage (Table VI).

**Table VI:** Performance of PPB Predicting Models for Specific Ionization States

	r^2^/Q^2^	RMSE	Coverage
Acid			
TS (5-FOLD CV)	0.61	0.20	-
EVS	0.72	0.17	
EVS (with two-class real-random classification AD)	0.73	0.17	0.96
Base			
TS (5-FOLD CV)	0.60	0.18	-
EVS	0.46	0.20	
EVS (with two-class real-random classification AD)	0.50	0.21	0.60
Neutral			
TS (5-FOLD CV)	0.70	0.18	-
EVS	0.47	0.19	
EVS (with two-class real random classification AD)	0.75	0.16	0.50
Zwitterion			
TS (5-FOLD CV)	0.64	0.18	-
EVS	0.46	0.21	
EVS (with two class real-random classification AD)	0.86	0.23	0.62

## Discussion

It is difficult to compare our results with literature models since they are often based on different datasets and different transformations are applied to the endpoint. To the best of our knowledge only few studies (3,42,43) used similar forms of pseudo-equilibrium constant for model derivation while nobody has used √*fu*. These models resulted in a r^2^ in internal and external validation often lower than 0.60, with the best model returning r^2^ =0.67 in external validation (42).

A larger number of models have been developed for predicting the percentage of chemicals bounded to plasma proteins (%PPB) (2,5,14,42,44–46). Recently Basant (45) reviewed literature models for %PPB and proposed a new model, returning very high performance in external validation (i.e., r^2^ greater than 0.90). A major limitation of this model was represented by the distribution of %PPB that was highly unbalanced towards higher values, leading to biased statistical performance. The use of pseudo-equilibrium constant instead of %PPB, as described in the work here presented, allows to overcome the risk of a biased validation.

In his studies on the Yamazaki dataset, Gleeson pointed out that PPB is closely related to both the ionization state and the liphophilicity of a molecule (3). Dealing with different representations of molecules (i.e., ionization states and tautomerism) is often a mandatory process especially when using ligand-receptor based models (47,48). Different SMILES representations of the same molecule lead to different descriptor values (49). In particular ionization state can influence a large block of descriptors, from charge-based descriptors to molecular properties. For example PPB is closely correlated with lipophilicity. If we compute the Pearson correlation between the Moriguchi octanol-water partition coefficient (MLOGP) calculated on neutralized SMILES of our TS and the same descriptor calculated on ionized SMILES, we get a value under 0.70.

As shown in Table VII, VSURF selection showed a certain degree of overlap in terms of selected descriptors between the logK and √fu predicting models. This is not unexpected because, although they are the result of different mathematical transformations, the two endpoints basically describe the same property, i.e. PPB. Consequently, the same properties are useful in both cases to explain the endpoint.

**Table VII:** List of Descriptors as Selected by VSURF Included in PPB Predictive Models

Common descriptors	Exclusive descriptors for LogK	Exclusive descriptors for √fu
ALOGP	nCsp2	CATS2D_01_LL
P_VSA_i_2	MLOGP2	nCar
MLOGP	GATS1i	SpMin1_Bh(i)
P_VSA_p_3	SpMax2_Bh(p)	Eta_betaP_A
C%	nBM	SM12_AEA(ri)
CATS2D_00_LL	MATS5e	nN+
Eta_betaP	AMW	
PCD	F01[C-N]	
Ui	T(O..O)	
N%	J_D/Dt	
C-024	SpMax_AEA(dm)	
CATS2D_00_PP		
totalcharge		

Several descriptors are related to lipophilicity (P_VSA_i_2, CATS2D_00_LL,CATS2D_01_LL, pMax2_Bh(p), MLOGP, MLOGP2, ALOGP), indeed it is well recognized that PPB is related to lipophilicity (50). In general, as compounds become more lipophilic, PPB becomes easier to predict, although some hydrophilic compounds have unexpected high PPB values (51). “Totalcharge” descriptor measures the sum of formal charges of each atom in a molecule. It is easy to understand that it is highly dependent on calculation of the correct ionization state of the molecule. For instance warfarin at pH 7.4 is a heterocyclic anion, and is known that albumin has specific binding sites to negatively charged hydrophobic compounds (2).

As shown in Table VIII the models fail in predicting compounds with the presence of charged N, that have been reported to be predicted correctly by other models, due to the high correlation of protein binding and LogP for these compounds (6).

**Table VIII:** List of Chemical Categories Showing a High Error in Prediction (Only Categories with a p <0.05 are Shown)

Name	Description	original dataset	Likelihood Ratio
Nq	quaternary N	Acid	7.50
N+	positively charged N	Acid	7.50
RCOOR	esters (aliphatic)	Base	1.85
OHt	tertiary alcohols	Base	1.98
RCONH2	primary amides (aliphatic)	CORAL	2.01
CH2RX	CH2RX	LogK	3.58
CONN	urea (-thio) derivatives	*√fu*	1.98
ArOH	aromatic hydroxyls	*√fu*	2.12
RCONHR	secondary amides (aliphatic)	*√fu*	5.12

**Table IX:** List of Chemical Categories with a Small Error in Prediction (Only Categories with a p <0.05 are Shown)

Name	Description	original dataset	Likelihood Ratio
Cq	total quaternary C(sp3)	Acid	7.50
Crq	ring quaternary C(sp3)	Acid	7.50
Cq	total quaternary C(sp3)	LogK	1.85
Beta-Lactams	Beta-Lactams	LogK	1.98
RSR	sulfides	LogK	2.01
Imidazoles	Imidazoles	*√fu*	3.58
Crq	ring quaternary C(sp3)	*√fu*	1.98
Cq	total quaternary C(sp3)	*√fu*	2.12
OHp	primary alcohols	*√fu*	5.12

Overall there is a clear predominance of good predictions for compounds with quaternary carbon atoms, like in branched alkanes that are usually highly liphophilic compounds (Table IX). The presence of descriptors like logP and number of aromatic carbons have a high specificity in predicting the interaction between imidazole and aminoacidic residues of albumin, like tryptophan (Trp), tyrosine (Tyr) and phenylalanine (Phe) (52) and might influence the predictivity of √*fu* model for imidazole category.

## Conclusion

In the present study, we derived new QSAR models predicting PPB. Mathematical transformations were applied to experimental data in order to obtain datasets suitable for modeling. Different combinations of descriptors and machine learning approaches were explored and applied to the endpoint.

SMILES using the ionization state did not make any significant contribution in model derivation compared to previous modeling efforts with similar algorithms (2), probably because some descriptors were not optimized for a correct interpretation of a charged compound (e.g. AlogP). Despite this, models still gave an acceptable result.

Performance in prediction confirmed the robustness of the derived models and their suitability for real-life purposes, i.e., screening chemicals for ADMET profiling.

## Electronic Supplementary Material

ESM 1 (XLSX 343 kb)

## ABBREVIATIONS

5-fold cv: 5 fold internal cross-validation
ACF: Atom centered fragment
AD: Applicability domain
CORAL: CORelation And Logic
CS: Calibration set
CW: Correlation weight
EVS: External validation set
*fu*: Fraction unbound
HA: Heuristic algorithm
ITS: Invisible training set
k-NN: k-nearest neighbor
LogK: Decimal logarithm of the pseudo constant derived from fu
LR: Likelihood ratio
MAE: Mean absolute error
PCA: Principal component analysis
PPB: Plasma protein binding
QSAR: Quantitative structure-activity relationship
RMSE: Root mean square error
SMILES: Simplified molecular input line entry system
SVM: Support vector machine
TS: Training set
VSURF: Variable selection (with) random forest

## References

[1] Zhivkova Z, Doytchinova I (2012). Quantitative structure—plasma protein binding relationships of acidic drugs. Journal of pharmaceutical sciences..

[2] Ghafourian T, Amin Z (2013). QSAR Models for the Prediction of Plasma Protein Binding. Bioimpacts..

[3] Gleeson MP (2007). Plasma protein binding affinity and its relationship to molecular structure: an in-silico analysis. J Med Chem..

[4] Martin YC, Abagyan R, Ferenczy GG, Gillet VJ, Oprea TI, Ulander J (2016). Glossary of terms used in computational drug design, part II (IUPAC Recommendations 2015). Pure and Applied Chemistry..

[5] Lambrinidis G, Vallianatou T, Tsantili-Kakoulidou A (2015). In vitro, in silico and integrated strategies for the estimation of plasma protein binding. A review. Adv Drug Deliv Rev..

[6] Hall LM, Hall LH, Kier LB (2009). Methods for predicting the affinity of drugs and drug-like compounds for human plasma proteins: a review. Current Computer-Aided Drug Design..

[7] L. L, Brunton PBAC, MD Bjorn Christian Knollmann, MD, PhD. Goodman & Gilman's. The pharmacological basis of therapy. 12th edition ed2011.

[8] Xue C, Zhang R, Liu H, Yao X, Liu M, Hu Z (2004). QSAR models for the prediction of binding affinities to human serum albumin using the heuristic method and a support vector machine. Journal of chemical information and computer sciences..

[9] Lexa KW, Dolghih E, Jacobson MP (2014). A structure-based model for predicting serum albumin binding. PloS one..

[10] Önlü S, Türker SM (2017). Impact of geometry optimization methods on QSAR modelling: A case study for predicting human serum albumin binding affinity. SAR and QSAR in Environmental Research..

[11] Obach RS, Lombardo F, Waters NJ (2008). Trend analysis of a database of intravenous pharmacokinetic parameters in humans for 670 drug compounds. Drug Metab Dispos..

[12] Collaborative Drug Discovery I. ChemCell - Cheminformatics Workflow Automation for Microsoft Excel. 2010.

[13] Obach RS, Lombardo F, Waters NJ. Trend analysis of a database of intravenous pharmacokinetic parameters in humans for 670 drug compounds. Drug Metabolism and Disposition. 2008. 10.1124/dmd.108.020479 18426954

[14] Yamazaki K, Kanaoka M (2004). Computational prediction of the plasma protein-binding percent of diverse pharmaceutical compounds. Journal of Pharmaceutical Sciences..

[15] Chemaxon. JChem for Office (Excel). JChem for Office. 17.22 ed2017.

[16] Kode. Dragon (Software for Molecular Descriptor Calculation) version 7.0. Kode srl; 2016.

[17] Kuhn M, Johnson K. Applied Predictive Modeling. 1 ed: Springer-Verlag New York; 2013. p. XIII, 600.

[18] Genuer R, Poggi J, Tuleau-Malot C. VSURF: Variable Selection Using Random Forests. 2016.

[19] Breiman L. Random forests. Machine learning. 2001;45(1):5-32.

[20] Berthold MR, Cebron N, Dill F, Gabriel TR, Kötter T, Meinl T (2009). KNIME-the Konstanz information miner: version 2.0 and beyond. AcM SIGKDD explorations Newsletter..

[21] Jaworska J, Nikolova-Jeliazkova N, Aldenberg T (2005). QSAR applicability domain estimation by projection of the training set descriptor space: a review. ATLA-NOTTINGHAM-..

[22] Netzeva TI, Worth AP, Aldenberg T, Benigni R, Cronin MT, Gramatica P (2005). Current status of methods for defining the applicability domain of (quantitative) structure-activity relationships. ATLA..

[23] Gadaleta D, Mangiatordi GF, Catto M, Carotti A, Nicolotti O (2016). Applicability domain for QSAR models: where theory meets reality. International Journal of Quantitative Structure-Property Relationships (IJQSPR)..

[24] Hastie T. Tibshirani, R. and Friedman, J.(2009): The elements of statistical learning. Data mining, inference, and prediction. Springer, New York, ISBN; 2008.

[25] Afantitis A, Melagraki G, Sarimveis H, Koutentis PA, Markopoulos J, Igglessi-Markopoulou O (2008). Development and evaluation of a QSPR model for the prediction of diamagnetic susceptibility. Molecular Informatics..

[26] Melagraki G, Afantitis A, Sarimveis H, Koutentis PA, Kollias G, Igglessi-Markopoulou O (2009). Predictive QSAR workflow for the in silico identification and screening of novel HDAC inhibitors. Mol Divers..

[27] Afantitis A, Melagraki G, Koutentis PA, Sarimveis H, Kollias G (2011). Ligand-based virtual screening procedure for the prediction and the identification of novel β-amyloid aggregation inhibitors using Kohonen maps and Counterpropagation Artificial Neural Networks. European Journal of Medicinal Chemistry..

[28] Melagraki G, Afantitis A, Sarimveis H, Igglessi-Markopoulou O, Koutentis PA, Kollias G (2010). In silico exploration for identifying structure–activity relationship of MEK inhibition and oral bioavailability for isothiazole derivatives. Chemical biology & drug design..

[29] Dimitrov S, Dimitrova G, Pavlov T, Dimitrova N, Patlewicz G, Niemela J (2005). A stepwise approach for defining the applicability domain of SAR and QSAR models. Journal of chemical information and modeling..

[30] Patlewicz G, Dimitrov SD, Low LK, Kern PS, Dimitrova GD, Comber MI (2007). TIMES-SS—a promising tool for the assessment of skin sensitization hazard. A characterization with respect to the OECD validation principles for (Q) SARs and an external evaluation for predictivity. Regulatory Toxicology and Pharmacology..

[31] Kühne R, Ebert R-U, Schüürmann G (2009). Chemical domain of QSAR models from atom-centered fragments. Journal of chemical information and modeling..

[32] Toropova AP, Toropov AA, Benfenati E, Leszczynska D, Leszczynski J (2010). QSAR modeling of measured binding affinity for fullerene-based HIV-1 PR inhibitors by CORAL. Journal of mathematical chemistry..

[33] Toropova AP, Toropov AA, Benfenati E, Gini G, Leszczynska D, Leszczynski J (2011). CORAL: quantitative structure–activity relationship models for estimating toxicity of organic compounds in rats. Journal of computational chemistry..

[34] Toropova A, Toropov A, Martyanov S, Benfenati E, Gini G, Leszczynska D (2012). CORAL: QSAR modeling of toxicity of organic chemicals towards Daphnia magna. Chemometrics and Intelligent Laboratory Systems..

[35] Advanced Chemistry Development I. ACD/labs. Toronto, ON, Canada 2010.

[36] Toropova AP, Toropov AA, Marzo M, Escher SE, Dorne JL, Georgiadis N (2018). The application of new HARD-descriptor available from the CORAL software to building up NOAEL models. Food and Chemical Toxicology..

[37] Toropov AA, Toropova AP (2017). The index of ideality of correlation: A criterion of predictive potential of QSPR/QSAR models?. Mutation Research/Genetic Toxicology and Environmental Mutagenesis..

[38] Toropova AP, Toropov AA (2017). The index of ideality of correlation: A criterion of predictability of QSAR models for skin permeability?. Science of the Total Environment..

[39] Toropov AA, Toropova AP, Marzo M, Dorne JL, Georgiadis N, Benfenati E (2017). QSAR models for predicting acute toxicity of pesticides in rainbow trout using the CORAL software and EFSA’s OpenFoodTox database. Environmental toxicology and pharmacology..

[40] Golbraikh A, Tropsha A (2002). Predictive QSAR modeling based on diversity sampling of experimental datasets for the training and test set selection. Mol Divers..

[41] Ferrari T, Cattaneo D, Gini G, Golbamaki Bakhtyari N, Manganaro A, Benfenati E (2013). Automatic knowledge extraction from chemical structures: the case of mutagenicity prediction. SAR and QSAR in Environmental Research..

[42] Zhu X-W, Sedykh A, Zhu H, Liu S-S, Tropsha A (2013). The use of pseudo-equilibrium constant affords improved QSAR models of human plasma protein binding. Pharmaceutical research..

[43] Rodgers SL, Davis AM, van de Waterbeemd H (2007). Time-series QSAR analysis of human plasma protein binding data. QSAR & Combinatorial Science..

[44] Votano JR, Parham M, Hall LM, Hall LH, Kier LB, Oloff S (2006). QSAR modeling of human serum protein binding with several modeling techniques utilizing structure− information representation. Journal of medicinal chemistry..

[45] Basant N, Gupta S, Singh K (2016). Predicting binding affinities of diverse pharmaceutical chemicals to human serum plasma proteins using QSPR modelling approaches. SAR and QSAR in Environmental Research..

[46] Saiakhov RD, Stefan LR, Klopman G (2000). Multiple computer-automated structure evaluation model of the plasma protein binding affinity of diverse drugs. Perspectives in Drug Discovery and Design..

[47] Natesan S, Subramaniam R, Bergeron C, Balaz S (2012). Binding affinity prediction for ligands and receptors forming tautomers and ionization species: inhibition of mitogen-activated protein kinase-activated protein kinase 2 (MK2). Journal of medicinal chemistry..

[48] Natesan S, Balaz S (2013). Rigorous Incorporation of Tautomers, Ionization Species, and Different Binding Modes into Ligand-Based and Receptor-Based 3D-QSAR Methods. Current pharmaceutical design..

[49] Gramatica P, Cassani S, Roy PP, Kovarich S, Yap CW, Papa E (2012). QSAR Modeling is not “Push a Button and Find a Correlation”: A Case Study of Toxicity of (Benzo-) triazoles on Algae. Molecular Informatics..

[50] Lázníček M, Lázníčková A (1995). The effect of lipophilicity on the protein binding and blood cell uptake of some acidic drugs. Journal of pharmaceutical and biomedical analysis..

[51] Croom E. Metabolism of xenobiotics of human environments. Progress in molecular biology and translational science. 112: Elsevier; 2012. p. 31-88. 10.1016/B978-0-12-415813-9.00003-9 22974737

[52] Jayabharathi J, Thanikachalam V, Perumal MV (2011). Mechanistic investigation on binding interaction of bioactive imidazole with protein bovine serum albumin—A biophysical study. Spectrochimica Acta Part A: Molecular and Biomolecular Spectroscopy..

